# *Fructus Ligustri Lucidi* in Osteoporosis: A Review of its Pharmacology, Phytochemistry, Pharmacokinetics and Safety

**DOI:** 10.3390/molecules22091469

**Published:** 2017-09-05

**Authors:** Beibei Chen, Lili Wang, Lin Li, Ruyuan Zhu, Haixia Liu, Chenyue Liu, Rufeng Ma, Qiangqiang Jia, Dandan Zhao, Jianzhao Niu, Min Fu, Sihua Gao, Dongwei Zhang

**Affiliations:** 1 Traditional Chinese Medicine School, Beijing University of Chinese Medicine, Beijing 100029, China; cbb8969@163.com (B.C.); lilin19930901@163.com (L.L.); zhuruyuan7@163.com (R.Z.); lhx8866_7@163.com (H.L.); marufeng5188@126.com (R.M.); jiaqiangqiang1234@163.com (Q.J.); niujianzhao@126.com (J.N.); 2 Chinese Material Medica School, Beijing University of Chinese Medicine, Beijing 100029, China; wangxiaoxing0108@163.com (L.W.); liuchenyue633@163.com (C.L.); 3 Diabetes Research Center, Beijing University of Chinese Medicine, Beijing 100029, China; bucmzhaodandan@163.com (D.Z.); gaosihua1216@163.com (S.G.); 4 The Research Institute of McGill University Health Center, Montreal, QC H4A 3J1, Canada; fumin1025@gmail.com

**Keywords:** *Fructus Ligustri Lucidi* (FLL), osteoporosis, pharmacology, phytochemistry, pharmacokinetics, safety

## Abstract

*Background*: *Fructus Ligustri Lucidi* (FLL) has now attracted increasing attention as an alternative medicine in the prevention and treatment of osteoporosis. This study aimed to provide a general review of traditional interpretation of the actions of FLL in osteoporosis, main phytochemical constituents, pharmacokinetics, pharmacology in bone improving effect, and safety. *Materials and Methods*: Several databases, including PubMed, China National Knowledge Infrastructure, National Science and Technology Library, China Science and Technology Journal Database, and Web of Science were consulted to locate publications pertaining to FLL. The initial inquiry was conducted for the presence of the following keywords combinations in the abstracts: *Fructus Ligustri Lucidi*, osteoporosis, phytochemistry, pharmacokinetics, pharmacology, osteoblasts, osteoclasts, salidroside. About 150 research papers and reviews were consulted. *Results*: FLL is assumed to exhibit anti-osteoporotic effects by improving liver and kidney deficiencies and reducing lower back soreness in Traditional Chinese Medicine (TCM). The data from animal and cell experiments demonstrate that FLL is able to improve bone metabolism and bone quality in ovariectomized, growing, aged and diabetic rats through the regulation of PTH/FGF-23/1,25-(OH)_2_D_3_/CaSR, Nox4/ROS/NF-κB, and OPG/RANKL/cathepsin K signaling pathways. More than 100 individual compounds have been isolated from this plant. Oleanolic acid, ursolic acid, salidroside, and nuzhenide have been reported to exhibit the anti-osteoporosis effect. The pharmacokinetics data reveals that salidroside is one of the active constituents, and that tyrosol is hard to detect under physiological conditions. Acute and subacute toxicity studies show that FLL is well tolerated and presents no safety concerns. *Conclusions*: FLL provides a new option for the prevention and treatment of osteoporosis, which attracts rising interests in identifying potential anti-osteoporotic compounds and fractions from this plant. Further scientific evidences are expected from well-designed clinical trials on its bone protective effects and safety.

## 1. Introduction

Osteoporosis is a kind of skeletal disorder characterized by reduced bone mass and deteriorated bone microarchitecture, which leads to compromised bone strength predisposing a person to an increased risk of fractures [1,2]. As the ageing population increases worldwide, the incidence of osteoporosis will increase further, which will lead to an estimated increase in the incidence of hip fractures around the world from 1.66 million in 1990 to 6.26 million in 2050 [3]. Osteoporosis causes a major public health and economic burden on global society because of the associated high morbidity, mortality and costs [3,4]. Therefore, there is an urgent need to find cost-efficient ways to prevent the development of osteoporosis. Traditional Chinese Medicines (TCMs) have been historically recorded as a major source of regimens for the prevention and treatment of fractures and joint diseases with relatively low cost, low risks of side effects and multiple target actions [5,6,7].

**Figure 1:**
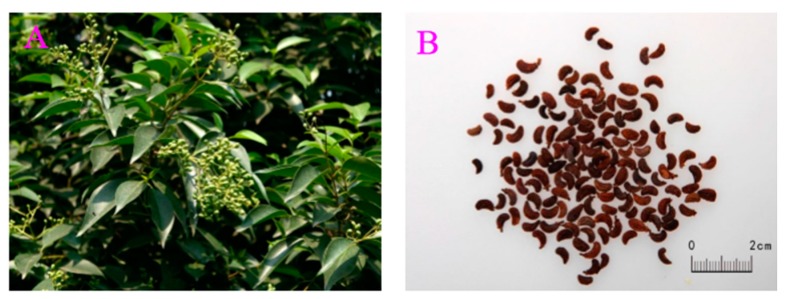
Representative images of *Ligustrum lucidum* Ait (**A**); and *Fructus Ligustri Lucidi* (**B**); which are used for isolating constituents for the treatment of osteoporosis. Pictures were kindly provided by Zexin Ma from the Chinese Medicine and Medica Museum, Beijing University of Chinese Medicine, China.

*Fructus Ligustri Lucidi* (FLL), known as Nvzhenzi in Chinese, is the dried mature fruit of *Ligustrum lucidum Ait*. (Oleaceae.) (Figure 1), and has been clinically used to treat osteoporotic bone pain and rheumatic bone for more than 1000 years [8,9,10]. It is mainly produced in Hu’nan, Sichuan, Jiangsu and Zhejiang provinces in China, and is harvested in winter by plucking the ripe fruits. FLL was first recorded in *Shen Nong*’s *Classic Materia Medica* (written during the period from 100 BCE to 200 CE), and labeled as a “top-tier” medicine (the ancient term “top-tier” was given to herbs without observable toxicity).

In the Chinese Pharmacopoeia (2015 version), FLL is recognized as an herb with healthy energy-supporting and kidney-liver tonifying effects. So far, FLL has been extensively studied in more than 3000 publications sourced from the Chinese National Knowledge Infrastructure (http://www.cnki.net/), National Science and Technology Library (http://www.nstl.gov.cn/), China Science and Technology Journal Database (http://en.cqvip.com/cstj.html), and approximately 120 from the PubMed (www.pubmed.gov) database. Emerging evidence suggests that FLL has many biological actions, including anti-osteoporosis [11], anti-oxidation [12], anti-diabetes [13], anti-ageing [14], anti-cancer [15], etc. Phytochemists have demonstrated that FLL contains more than 100 chemical components, such as terpenoids, flavonoids, phenylethanoid glycosides, phospholipids, polysaccharides, etc. [15]. Here, we review the recent advances of FLL in the management of bone metabolism and quality in preclinical experiments, phytochemistry, pharmacokinetics as well as safety. Firstly, we summarize the interpretation of the actions of FLL in TCM, which helps to understand anti-bone resorption and pro-anabolic activities of this herb in the management of osteoporosis.

## 2. The Bone-Protective Actions of *Fructus Ligustri Lucidi* in the Theory of Traditional Chinese Medicine

In TCM textbooks, FLL is recorded as an herb with a bitter and sweet flavor (here the word flavor not only reflects the taste of the herb, but also corresponds to a physiological response and organ target based on ancient observations) as well as cool property (the property reflects the combined effects of the Chinese medicine felt by a patient and the outcome of the treatment) [16,17]. According to the theory of meridian tropism [18], which describes the in vivo targeting of a Chinese medicine to certain organs, FLL can be distributed to liver and kidney. In TCM, bitter flavor has the function of “clearing heat (anti-inflammation and inhibition of infections)” and “drying (eliminating dampness)” as well as “insisting (improving the functions of kidney and liver)”. Further, sweet flavor has the function of tonifying and calming as well as reducing irritation and generating fluids. Therefore, FLL has the function of nourishing the liver and kidney as well as blackening hair and improving eyesight. FLL has been historically used to improve *Yin* deficiency of liver and kidney manifested with symptoms of blurred and dark vision, and with decreased visual acuity and early hair whitening, and to relief lower back soreness. The recommended daily dosage for FLL is 6 to 15 g. In addition, FLL can be processed with rice wine to improve its effects on kidney and liver in TCM clinical practice.

In addition, osteoporosis in TCM is recognized as “bone atrophy”, “bone loss” or “bone rheumatism” [19]. The main etiology and pathogenesis of osteoporosis is attributed to the deficiencies in kidney, liver and spleen as well as blood stasis [20]. Meanwhile, FLL has the ability of improving liver and kidney deficiencies and reducing lower back soreness. Thusly, FLL can be clinically used to treat osteoporosis in prescriptions containing other herbs, such as *Herbal Epimedii* [21], *Fructus Psoraleae* [22], *Herba Ecliptae* [23] and *Puerariae radix* [24,25,26].

## 3. Phytochemistry of *Fructus Ligustri Lucidi*

**Figure 2:**
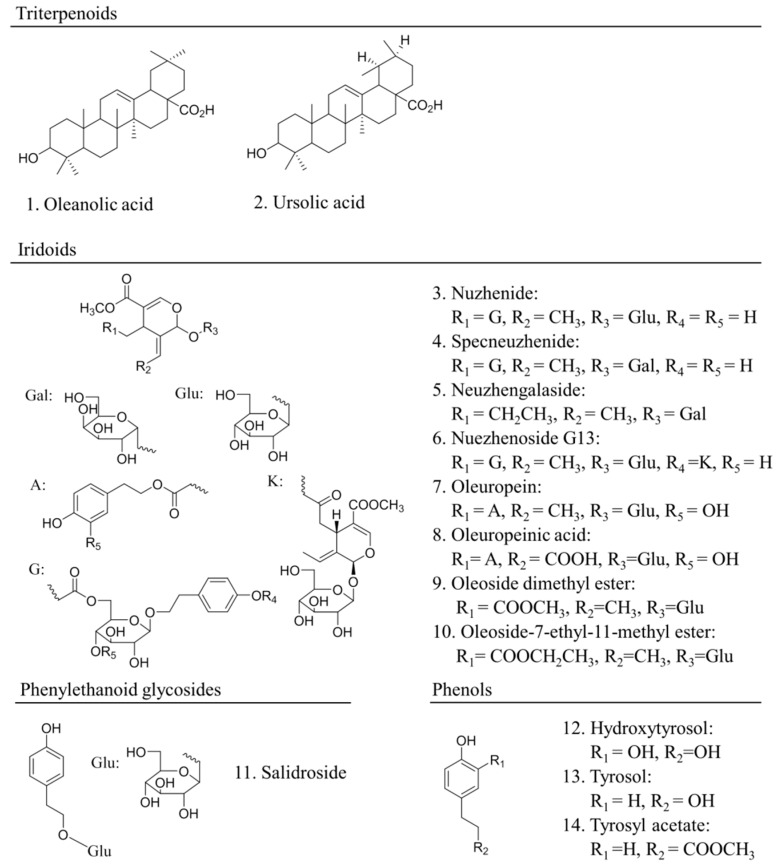
The chemical structures of the main active ingredients isolated from *Fructus Ligustri Lucidi*: (**1**) oleanolic acid; (**2**) ursolic acid; (**3**) nuzhenide; (**4**) specneuzhenide; (**5**) neuzhengalaside; (**6**) nuezhenoside G13; (**7**) oleuropein; (**8**) oleuropeinic acid; (**9**) oleoside dimethyl ester; (**10**) oleoside-7-ethyl-11-methyl ester; (**11**) salidroside; (**12**) hydroxytyrosol; (**13**) tyrosol; (**14**) tyrosyl acetate.

Numerous molecular constituents of FLL have been isolated from the plant during the past decades. Currently, more than 100 compounds, including terpenoids, flavonoids, phenylethanoid glycosides, phospholipids, polysaccharides, volatile components, amino acids, and others [27], have been identified from this plant according to the Chinese Academy of Sciences Chemistry (www.organchem.csdb.cn) and Chinese Herbal Drug Databases. The main ingredients of this herb are shown in Figure 2 So far, it has been demonstrated that triterpenoids and phenylethanoid glycosides are two major types of constituents present in high content and responsible for the main pharmacological actions for FLL [28].

Both oleanic acid (OA) and ursolic acid (UA) are two representative triterpenoids with certain solubility in water, methanol, ethanol, 2-propanol, and ethyl acetate [29,30,31,32]. These two compounds have been evidenced to exhibit a variety of activities, including stabilizing liposomal membranes [33], anti-cancer [34], anti-diabetes [35,36], anti-inflammation [37], anti-oxidation [2], anti-osteoporosis [38,39], etc. In addition, OA is the most abundant potent triterpenoid in FLL with a content ranging from 7.37 to 13.3 mg/g [40]. However, this content of OA may vary depending on the different parts of fruits, regions, harvest seasons, and processing methods [27,28,40,41].

In addition to OA and specnuezhenide [28], salidroside is one of major reference compounds and most studied phenylethanoid glycosides [30]. The content of salidroside in the crude FLL power determined by HPLC ranges from 5.7 to 6.9 mg/g [42]. Currently, there are more than 400 publications investigating the biological activities of salidroside, including anti-diabetes [43], anti-obesity [44], anti-cancer [44,45], anti-osteoporosis [46], anti-arthritis-induced brain cognition deficits [47], anti-hypertension [48,49], and cardioprotective effects [50].

The iridoids in FLL mainly include specnuezhenide, nuezhenide, ligustroflavone, neuzhengalaside, nuezhenoside G13, oleuropein, oleuropeinic acid, oleoside dimethyl ester and other compounds [27,28,30]. These compounds have been demonstrated to possess anti-oxidant effects [51,52,53]. Specnuezhenide, with a content of 0.70–3.06%, is one of main reference compounds for FLL in the 2015 Chinese Pharmacopoeia [54].

In addition, with the development of newer analytic techniques, it can be expected that new constituents of FLL will be discovered, which will contribute to fully understanding the pharmacological effects of this herb, and offer new chemical scaffolds for the development of novel anti-osteoporosis drugs.

## 4. Pharmacokinetic of *Fructus Ligustri Lucidi*

The absorption, distribution, metabolism and elimination of an herb after oral administration significantly affect its biological actions. So far, there are a few papers that study the pharmacokinetic profiles of FLL, mainly focused on the metabolic properties of salidroside in rats.

Peng et al. studied the pharmacokinetic parameters of salidroside in normal rats. The results revealed that the C_max_ (the maximum plasma concentration during the whole process), T_max_ (the time corresponding to the maximum plasma concentration), AUC_0-t_ (area under the plasma concentration-time curve from zero (0) h to time (t)), MRT (the average time drug molecules are retained in vivo after rapid intravenous infusion), T_1/2_ (the time taken for a drug to clear from the highest concentration to half of this level) and K (drug clearance rate constant from the central chamber to the outside in the one-compartment model) were 19.29 µg/mL, 0.5 h, 21 μg/mL·h, 3.43 h, 3.28 h, 0.21, respectively [55]. Further, they studied the pharmacokinetic parameters of salidroside in a rat model of migraine, where the results revealed that the C_max_, T_max_, AUC_0-t_, MRT, T_1/2_ and K were 20.71 µg/mL, 0.5 h, 28.75 μg/mL·h, 2.36 h, 1.44 h, 0.48, respectively [56]. In addition, in a rat model of insomnia, the C_max_, T_max_, AUC_0-t_, MRT, T_1/2_ and K of salidroside were 20.24 µg/mL, 0.5 h, 28.31 μg/mL·h, 2.80 h, 2.53 h, 0.27, respectively [57]. Interestingly, C_max_ and T_max_ exhibit no differences in the different rat models, however, AUC_0-t_ was increased in the rat model of migraine or insomnia, suggesting that the bioavailability of salidroside was improved in response to external insults. However, further investigations are still needed to confirm the tissue distributions of FLL after absorption.

Interestingly, the investigators found that plasma concentration of tyrosol is too low to be detected in the normal rats using current analytical technology [55]. However, in the rat model of migraine, the plasma concentration of tyrosol was significantly increased and the corresponding pharmacokinetic parameters were as follows: C_max_ (0.76 μg/mL), T_max_ (0.75 h), AUC_0-t_ (0.87 μg/mL·h), MRT (1.36 h), T_1/2_ (0.74 h) and K (0.94) [56]. Further, the C_max_ and T_max_ of tyrosol were 0.49 µg/mL and 0.75 h, respectively, in insomniac rats. The maximum plasma concentration of tyrosol/salidroside was 3.90/100, in comparison to 2.99/100 in the normal rats. The tyrosol was detectable in the plasma after 15 min till 1 h after administration [57]. The results further indicated that the plasma concentration of tyrosol is markedly increased during migraine attacks. Also, tyrosol and salidroside may exhibit synergistic effects in response to external insults.

## 5. Anti-Bone Resorption and Pro-Anabolic Activities of *Fructus Ligustri Lucidi* in Bone Metabolism and Bone Quality

### 5.1. Effects of Fructus Ligustri Lucidi on Bone Metabolism and Quality in Animal Experiments

So far, the bone protective effect of FLL has been investigated in ovariectomized (OVX), aged, diabetic and growing rats. We summarize the possible applications of this herb involved in regulating bone metabolism and improving bone quality in preclinical studies shown in Table 1. In 2006, Zhang et al. were the first to report that FLL could improve bone turnover and calcium (Ca) balance in OVX rats [58]. In this experiment, 550 mg/kg/d of FLL (dried extract powder) were used to treat OVX rats for 14 weeks (from the 5th week to the 18th week after OVX). The results indicated that FLL was able to suppress an increase in serum levels of osteocalcin (OCN) and urinary deoxypyridinoline (DPD), and improve Ca metabolism by increasing intestinal Ca absorption, and inhibiting urinary Ca excretion in OVX rats.

In addition, Zhang et al. studied the effect of FLL ethanol extracts (700 mg/kg/d) on OVX rats fed with different levels of Ca diets for 12 weeks [59]. The results revealed that FLL increased bone mineral density (BMD) and biomechanical strength in the tibia diaphysis, and improved bone mineral content (BMC) at both tibia and femoral diaphysis and lumbar vertebra in rats on either low (0.1%) or medium (0.6%) Ca diet and FLL improved bone mass and inhibited bone loss of cortical and trabecular bone in OVX rats on high-Ca diet.

Further, Zhang et al. found that a combination of FLL ethanol extract with medium or high (1.2%) Ca diet increased serum parathyroid hormone (PTH) and 1,25-dihydroxyvitamin D3 [1,25-(OH)_2_D_3_] levels, and upregulated duodenal mRNA expression of CaBP-9k and VDR as well as renal mRNA expression of Ca-binding protein (CaBP)-28k in aged female SD rats [68]. It is known that Ca metabolism is mainly controlled by PTH, 1,25-(OH)_2_D_3_, vitamin D-dependent CaBP, and calcitonin (CT) [69]. PTH and CT are complementary hormones involved in maintenance of Ca metabolism and improvement of bone quality [70]. PTH enhances 1,25-(OH)_2_D_3_ production and vitamin D receptor (VDR) expression, which further contributes to the absorption of Ca in the intestine via regulating the expression of CaBP-9k and CaBP-28k [71,72].

Furthermore, it is known that 1,25-(OH)_2_D_3_ is mainly produced in the renal proximal tubules, and activated by 1α-hydroxylase (1-OHase), which up-regulates VDR expression [73,74]. 1,25-(OH)_2_D_3_, together with VDR, enhances subsequent CaBP-9k expression and Ca absorption rate [68]. Dong et al. found that FLL treatment (700 mg/kg) for 12 weeks increased the circulating levels of 1,25-(OH)_2_D_3_ by stimulating renal 1-OHase and VDR expression in OVX rats [60]. The results were also demonstrated by the addition of FLL ethanol extract (100 μg/mL) into rat primary proximal tubule cells for 48 h. Further, Feng et al. [61] demonstrated that FLL ethanol extract (0.65% and 0.90% in diet) improved both Ca absorption and retention rate through elevating serum levels of 1,25-(OH)_2_D_3_, and up-regulating the transcriptions of renal 1-OHase, duodenum VDR, CaBP-9k, and transient receptor potential vanilloid 6 (TRPV6) in growing male SD rats, which resulted in an improvement of BMD and bone microarchitecture as well as bone mechanical properties, suggesting that FLL ethanol extract improved bone properties by activating 1,25-(OH)_2_D_3_-dependent Ca transport in the kidneys and duodenum. In addition, Guo et al [63] demonstrated that FLL water extract (3.5/kg) treatment for 12 weeks increased serum Ca and P content, and reduced urinary Ca and P excretion in OVX rats. The improvement of FLL water extract on Ca and P metabolism was also confirmed by Cheng et al’s observation [64]. In this study, OVX rats were administrated with FLL water extract for 26 weeks, with the effective doses were demonstrated at 9 and 4.5 g/kg, respectively.

In order to further reveal the active ingredients which are responsible for its beneficial effects on Ca balance and bone properties, different fractions in FLL, including FLL ethanol extract (FLL-EE), ethyl acetate-soluble fraction of EE (FLL-EAF), and water-soluble fraction of EE (FLL-WF), were administrated to 4-month-old mature female SD rats for 12 weeks with low (0.1% Ca), medium (0.6% Ca), and high (1.2% Ca) dietary levels, respectively [65]. The results demonstrated that both FLL-WF and FLL-EE exhibited a substantial effect on Ca balance by inhibiting urinary and fecal Ca excretion. FLL-WF treatment improved Ca metabolism in rats fed with medium or high dietary Ca diet, and led to a slight increase in serum PTH levels accordingly. However, FLL-WF did not alter BMD or BMC in the tibias of the rats fed with different dietary Ca in mature female SD rats. Therefore, it may be concluded that FLL-WF was main fraction that contributed to the positive actions of FLL on Ca balance. However, further studies are still needed to evaluate the effects of different fractions and compounds on osteoporotic animal models, and to elucidate its involvement in regulation of 1,25-(OH)_2_D_3_ and PTH production.

Accumulating evidences have shown that type 1 diabetes is associated with higher fracture risk and lower bone quality [75,76,77,78,79]. Diabetes may affect bone health by increasing Ca excretion and bone loss [79,80,81]. Using streptozotocin (40 mg/kg for 5 consecutive days) treated DBA/2J mice, Zhang et al. [66] demonstrated that FLL-WF inhibited hypercalciuria and improved trabecular bone microstructures by increasing serum levels of PTH and fibroblast growth factor-23 (FGF-23), and upregulating mRNA expression of TRPV6 and CaBP-9k in the duodenum as well as decreasing mRNA and protein expression of calcium-sensing receptor (CaSR) in the kidney of diabetic mice. It is well known that PTH stimulates transcription and secretion of FGF-23, which further increases circulating Ca level [82,83,84]. Additionally, CaSR serves as a sensor controlling extracellular calcium homeostasis and may have an inhibitory effect on PTH secretion [85].

The osteoprotegerin (OPG)/receptor activator of nuclear factor κB ligand (RANKL)/receptor activator of nuclear factor κB (RANK) signaling system is evidenced to play an important role in regulating osteoclastogenesis and bone mass [86]. An increased RANKL/OPG ratio contributes to osteoclast formation and bone resorption [87]. Lyu et al. [67] demonstrated that treatment with FLL ethanol extract (0.40, 0.65 and 0.90% in diet) for 16 weeks dose-dependently decreased the ratios of RANKL/OPG mRNA in the tibias of growing female rats, which led to an increase in femoral BMD and microstructure and biomechanical properties (ultimate load and ultimate deformation).

Oxidative stress also contributes to the development of osteoporosis through increasing osteoclastogenesis and inhibiting osteoblastogenesis [88]. Our group demonstrated that FLL aqueous extract improved redox homeostasis evidenced by increasing the levels of total antioxidant capacity and nitric oxide as well as decreasing the levels of malondialdehyde and 8-hydroxy-desoxyguanosine in serum, tibias, and uteri of OVX rats [62]. The underlying mechanism may be owed to the potential of FLL in improving the NADPH oxidase 4 (Nox4)/ROS/NF-κB signaling pathway via downregulating the expression of Nox4, NF-κB-p65, NF-κB-pp65, and IκBα, as well as increasing Bcl-2 expression and preventing cytoplasmic release of mitochondrial cytochrome C in the tibias and femurs, which benefits for improving bone microstructure and cortical bone thickness in OVX rats.

**Figure 3:**
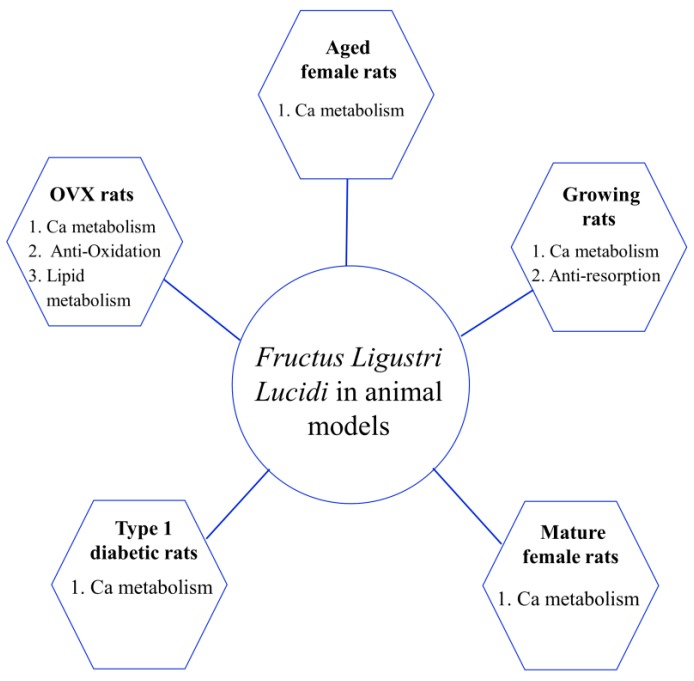
*Fructus Ligustri Lucidi* exhibited beneficial effects on bone metabolism in different animal models through improving Ca metabolism, reducing oxidative stress, regulating lipid metabolism and inhibiting bone resorption. Notes: Ca, calcium; OVX, ovariectomized.

Osteoporosis may lead to the disorders of lipid metabolism [89]. Our group further revealed that FLL water extract (3.5 g/kg) treatment for 12 weeks beneficially increased the serum level of high density lipoprotein, and decreased the serum levels of low density lipoprotein, total cholesterol and triglyceride in OVX rats [63]. In addition, FLL water extract (3.5 g/kg) has the capacity of improving collagen metabolism evidenced by increasing the serum levels of collagen I amino terminal peptide, and decreasing the serum levels of collagen I carboxyl terminal peptide and urine DPD in OVX rats [63]. Taken together, the mechanisms underlying the bone improvement effect of FLL may be attributed to an improvement of Ca and lipid metabolism and bone resorption as well as an inhibition of oxidation in different rat models (Figure 3). FLL-WF and FLL-EE are currently regarded as the material basis supporting the anti-osteoporotic effect of this herb. However, further isolation of the active fractions of FLL extract and its possible mechanism is still expected, which will contribute to fully understanding this traditional herb and developing new anti-osteoporosis drugs.

### 5.2. Effects of Fructus Ligustri Lucidi on Osteoblasts

Osteoblasts are derived from mesenchymal stem cells, which synthesize components of bone organic matrix such as collagen, OCN and osteopontin [90]. The insufficiency in the number of osteoblasts and the decrease in osteoblasts activities may impair bone remodeling and further result in osteoporosis [91]. In order to evaluate the effect of FLL on osteoblasts differentiation and maturation, different doses of FLL ethanol extract (0.1–100 μg/mL) were incubated with MC3T3-E1 cells [67] and UMR-106 cells [59]. The results from alkaline phosphatase (ALP) activity assays revealed that FLL ethanol extract dose-dependently increased MC3T3-E1 cells differentiation. In addition, alizarin red staining revealed that FLL ethanol extract dose-dependently increased UMR-106 cells mineralization. Further, FLL ethanol extract (1–100 μg/mL) contributed to an increase of extracellular Ca and P levels with a maximum concentration at 10 μg/mL. Real-time PCR results further demonstrated that FLL ethanol extract (10, 25 and 50 μg/mL) dose-dependently increased mRNA expression of ALP and bone sialoprotein and OCN. In addition, FLL treatment (10, 25 and 50 μg/mL) dose-dependently decreased the mRNA relative ratio of RANKL/OPG in MC3T3-E1 cells, which contributes to suppress osteoclastogenesis and subsequent bone resorption [92]. Together, the results suggest that FLL treatment is able to promote MC3T3-E1 cell differentiation and mineralization, and to inhibit osteoclasts activation.

In addition, the water extract of FLL also shows positive effects on osteoblastogenesis in vitro. Li et al. [93] found that water extract of FLL (10^−2^ and 10^−5^ mg/mL) could promote MC3T3-E1 cells proliferation and differentiation. In addition, 10^−5^ mg/mL of FLL water extract also increased the relative ratio of OPG/RANKL in MC3T3-E1 cells. The underlying mechanism governing the effect of FLL on osteoblasts may partly rest on regulation of the MAPK and Akt signaling pathways through increasing the phosphorylation of ERK, JNK, p38 and Akt according to western blotting and ELISA assays.

Furthermore, the ingredients of FLL have been isolated and characterized using bioactivity assays [51]. The results demonstrated that compounds isolated from FLL [hydroxytyrosol (1 μg/mL), salidroside (10 μg/mL), nuzhenide (10 μg/mL), and nuezhenoside G13 (10 and 50 μg/mL)] enhanced ALP activity in UMR-106 cells and oleoside dimethyl ester (1–10 μg/mL), oleoside-7-ethyl-11-methyl ester (10 μg/mL), and nuzhenide (1–50 μg/mL) could stimulate UMR-106 cell proliferation. The results suggest that nuzhenide could promot osteoblast differentiation and proliferation. Since both nuzhenide and nuezhenoside G13 contain a salidroside moiety, it is reasonable to postulate that salidroside may be a pharmacophore responsible for osteoblastogenesis in the constituents of FLL.

Eliminating oxidative stress also benefits the growth of osteoblasts [94,95,96]. In an experiment performed by Chen et al. [51] demonstrated that tyrosol, hydroxytyrosol, salidroside, nuzhenide, and nuezhenoside G13 derived from FLL exerted antioxidant activities using the ferric reducing antioxidant power, total antioxidant capacity, and radical 2,2-diphenyl-1-picrylhydrazyl scavenging activity assays in vitro.

In addition, estrogen receptor (ER) α/β-mediated estrogen response element (ERE) gene transcription positively contributed to osteoblastogenesis [96,97]. Using Hela cells that co-transfected with expression vector pClneo-hERα or pIRES-hERβ, pGL3-5xERE-luciferase reporter plasmid, and pSV-β-galactosidase plasmid, FLL water extract has been demonstrated to increase ERα and ERβ expression [51]. FLL water extract exhibited more potency in activating ERβ than ERα. Furthermore, Wang et al. [98] found that the stimulatory effect of FLL water extract on ALP activity in UMR-106 osteoblasts could be blocked by the addition of ER antagonist (ICI182780). Indeed, it is not surprising that trace amount of isoflavones (apigenin, luteolin, quercetin, etc) in FLL may be a source of estrogenic activity [99].

### 5.3. Effects of Fructus Ligustri Lucidi on Osteoclasts 

Osteoclasts are derived from the hematopoietic lineage and mainly responsible for bone resorption during the development of osteoporosis [100]. Upon stimulation by RANKL and macrophage colony-stimulating factor, the precursors of osteoclasts became mature and activated. Using RAW264.7 murine monocyte/macrophage cells, Xu et al. found that FLL extract and its two primary components OA and UA inhibited osteoclasts differentiation and bone resorption through suspending RANKL induced osteoclasts formation and subsequent tartrate resistant acid phosphatase (TRAP) activity [101]. Additionally, treatment with FLL ethanol extract, OA and UA also suppressed RANKL-induced osteoclastogenesis by reducing mRNA levels of tumor necrosis factor receptor associated factor 6, nuclear factor of activated T cell-c1(NFATc1) and c-Fos, as well as TRAP, cathepsin K and matrix metalloproteinase 9 in RAW264.7 cells. However, further studies are still needed to explore the precise mechanisms of FLL on osteoclastogenesis in animal models and clinical trials as well.

**Figure 4:**
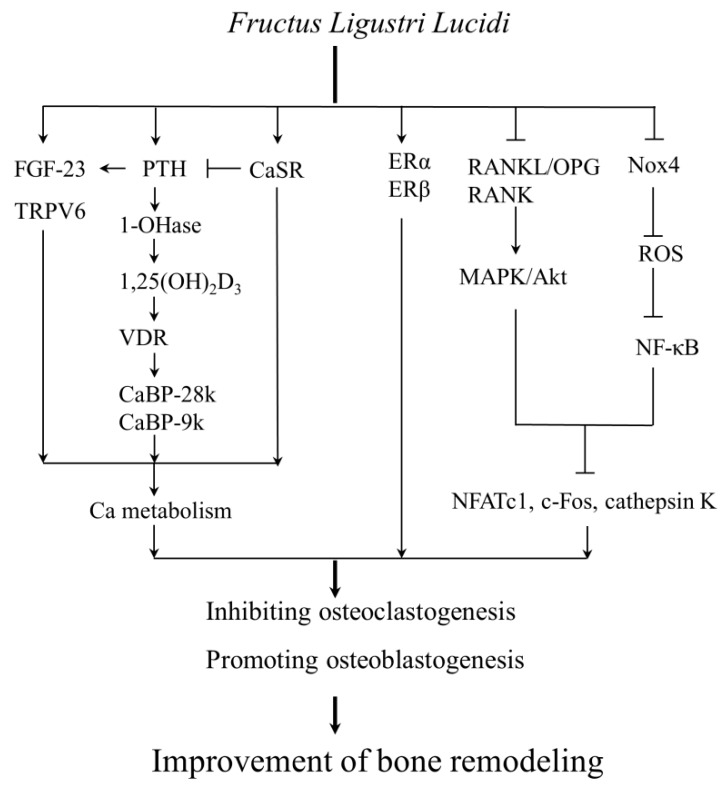
The revealed mechanisms targeted by *Fructus Ligustri Lucidi* in management of bone remodeling*. Fructus Ligustri Lucidi* improves Ca balance through upregulating the levels of PTH, 1-OHase, VDR, 1,25(OH)_2_D_3_, FGF-23, CaBP-9k, CaBP-28k, and TRPV6, and deregulating the levels of CaSR. In addition, *Fructus Ligustri Lucidi* inhibits osteoclastogenesis and promotes osteoblastogenesis through regulating Nox4/ROS/NF-κB, and suppressing RANKL/OPG/cathepsin K via regulating MAPK/Akt pathways. Further, *Fructus Ligustri Lucidi* may also play a role in the regulation of ERα and ERβ expression. Notes: 1,25-(OH)_2_D_3_, 1,25-dihydroxyvitamin D3; 1-OHase, 1α-hydroxylase; Akt, protein kinase B; CaBP-28k, Ca-binding protein-28k; CaBP-9k, Ca-binding protein-9k; CaSR, calcium-sensing receptor; ERα, estrogen receptor α; ERβ, estrogen receptor β; FGF-23, fibroblast growth factor-23; MAPK, mitogen-activated protein kinase; NFATc1, nuclear factor of activated T cell-c1; NF-κB, nucleic factor kappa-B; Nox4, NADPH oxidase 4; OPG, osteoprotegerin; PTH, parathyroid hormone; RANKL, receptor activator of nuclear factor κB ligand; ROS, reactive oxygen species; TRPV6, transient receptor potential vanilloid 6; VDR, vitamin D receptor.

In summary, in vivo and ex vivo experiments demonstrate that FLL improves bone microstructures and bone quality through improving Ca metabolism via increasing the levels of PTH, FGF-23, 1-OHase, 1,25-(OH)_2_D_3_ and VDR, and decreasing expression of CaSR, as well as exhibiting antioxidant effect via Nox4/ROS/NF-kB signaling pathway, along with regulating lipid metabolism in osteoporotic, diabetic, growing and aged rats (Figure 4). In addition, FLL promotes osteoblastogenesis and inhibits osteoclastogenesis through regulating OPG/RANKL ratio via controlling MAPK and Akt signaling pathways. Further, FLL exhibits estrogen-like effect in vitro and in vivo. Luckily, FLL does not show detrimental stimulatory effects on the uterus in the management of osteoporosis so far. The anti-osteoporotic effects of different fractions of FLL have been evaluated. And the ethanol extract of FLL and its water extract may be the biological fractions against osteoporosis. And nuzhenide, salidroside, and OA are demonstrated to be the active compounds for osteogenesis in FLL. However, FLL water extract also exhibited anti-osteoporotic effect in animal and cell models. Therefore, further studies are needed to figure out the real contributions of each fraction under the same preparation.

## 6. The Toxicity of *Fructus Ligustri Lucidi*

The toxicity of TCM herbs has attracted rising attention in the clinical trials [81,102,103]. However, it must be emphasized that the isolated chemicals and the extracts from the herbs are not identical to the original herbs or formulas, while the traditional properties and indications of TCM herbs and formulas are the validated source for interpreting and extrapolating toxicity assessments [103,104].

It is generally agreed that FLL is well tolerated in human and animals with a high margin of safety. One experiment performed by Dong et al. demonstrated FLL had no toxicity to mice and rats [105]. In this experiment, the supercritical carbon dioxide (CO_2_) extract of FLL was orally administrated to female and male mice for 7 days at the doses of 11, 13 and 15 g/kg, respectively. No mice were found to be abnormal or died by the end of the experiment. Further, the supercritical CO_2_ extract of FLL (3.75, 2.25, and 0.75 g/kg) was orally administrated to the female and male rats for 30 days. At the end of study, all the rats appeared normal physical activity, feed intake, body weight, and fecal characteristics. In addition, the results from the hematological examination and pathological observation presented no differences between groups. Therefore, the authors concluded that FLL was not toxic under normal feeding. In addition, the authors investigated the genotoxicity of FLL in mice by a micronucleus test [106]. Different doses of the supercritical CO_2_ extract of FLL (1.88, 3.75, and 7.50 g/kg) were orally given to female and male mice twice (at an interval of 24 h), the mice were sacrificed and samples were obtained 24 h after the second time. The results demonstrated that FLL treatment did not induce significant differences in bone marrow micronucleus examination, indicating that FLL has no observable genotoxicity.

Further, Xu et al. [107] conducted additional experiment to observe the acute toxicity of FLL in the broilers. In this study, 15 g/kg of the supercritical CO_2_ extract of FLL were repeatedly administrated intragastrically to broilers three times in 12 h. Then the broilers were kept for a further 14 days to observe any possible toxic reactions. Finally, the investigators did not find significant differences in body weight gains, physical appearance, and histopathological changes in the broilers, suggesting that FLL has no toxicity at the given dose with the maximum tolerable dose of 45.0 g/kg in the broilers.

Additionally, safety evaluations of FLL were conducted in rabbits and mice [108,109]. Five mg of fresh ethyl acetate extract of FLL leaves were injected into the mice by a tail intravenous route, and no mortality or obvious abnormality was observed after 24 h in the acute toxicity study. In another subacute toxicity study, rabbits were injected with fresh total ethyl acetate extract of FLL leaves (50 mg/kg/d) for 6 and 12 weeks. The results revealed that there was no abnormality in histopathological changes and no mortality occurred. Further, Ju et al. [53] found that ethyl acetate fraction of FLL had the capacity of protecting from oxidative damage in hydrogen peroxide-treated SH-SY5Y cells via improving intracellular antioxidant enzymes, and reducing lipid peroxidation level and caspase-3 activity.

In clinical trials, FLL treatment has the potential to cause thirst, dizziness, slight abdominal pain, and diarrhea [108]. However, these symptoms will disappear after cessation of using the herb. In a word, acute and subacute studies as well as clinical trials support the notion that FLL has no toxicity in animal experiments and human clinical trials at doses of no more than the acceptable daily intake. Nevertheless, caution must be exercised when FLL is taken at high and non-nutritional doses. Long-term toxicity studies of FLL are still needed to further evaluate the safety of this herb.

## 7. Conclusions and Outlook

FLL has been traditionally co-prescribed with other herbs to prevent and treat osteoporosis in TCM clinical trials. The results from animal and cell experiments also support the notion that FLL could improve the bone remodeling and quality in OVX, diabetic, growing and aged rats. The underlying mechanisms may be attributed to its regulations of PTH/FGF-23/1,25-(OH)_2_D_3_/CaSR, Nox4/ROS/NF-κB, and OPG/RANKL/cathepsin K signaling pathways. FLL also shows estrogen-like effects but without obvious detrimental stimulatory effects in the uterus. However, further studies are still expected to discover the precise mechanism of multi-target effects that FLL may harbor via using different osteoporotic animal models.

The phytochemical properties of FLL and its ingredients have also been well studied. Among over 100 structurally characterized compounds, terpenoids and phenylethanoid glycosides are the two major classes of constituents with diverse biological activities. Meanwhile, different fractions of FLL were employed to evaluate its anti-osteoporotic effects. The ethanol extract of FLL and its water extract have great potential in improving bone quality in osteoporotic animal models. Further studies on the pharmacokinetics of FLL will contribute to identify main active compounds and further provide a pharmaceutical core for developing new anti-osteoporosis drugs.

FLL is traditionally recognized as the “top-tier” herb without observable safety reactions in acute and subacute toxicity studies. However, long-term toxicity studies are further needed to verify the safety of this herb for continuous consumption in chronic and progressive bone diseases, which will benefit for developing new edible and medicinal herbs. In addition, prospective and well-designed clinical trials are also required to further verify the contributions of FLL in the management of the patients with osteoporosis. Combining the advances in TCM theory, phytochemistry, pharmacokinetics, pharmacology and safety studies, FLL supplementation will offer a new therapeutic promise in preventing the development of osteoporosis. To this end, further scientific evidences are required from prospective and well-designed clinical trials on its anti-osteoporosis effects and safety.

## Figures and Tables

**Table 1:** Animal models employed in studying the effect of *Fructus Ligustri Lucidi* on bone remodeling.

Animal Models	Fractions or Components of FLL	Dosage and Route	Duration of Treatment	Refs.
OVX rats	FLL extract	Oral gavage; 550 mg/kg/d	14 weeks	[58]
OVX rat	FLL ethanol extract	Oral gavage; 700 mg/kg/d	12 weeks	[59]
OVX rats	FLL ethanol extract	Oral gavage; 700 mg/kg/d	12 weeks	[60]
Male growing rats	FLL ethanol extract	0.40, 0.65 and 0.90% FLL extract with AIN-93G	4 months	[61]
OVX rats	FLL water extract	Oral gavage; 3.5 g/kg/d	12 weeks	[62,63]
OVX rats	FLL ethanol extract	Oral gavage; 9, 4.5, and 2.25 g/kg/d	26 weeks	[64]
Female mature rats	FLL ethanol extract (EE), ethyl acetate-soluble fraction of EE (EAF), water fraction of EE (WF)	Oral gavage; 700, 574 and 126 mg/kg/d	12 weeks	[65]
Male type 1 diabetic DBA/2J mice	water fraction of FLL ethanol extract	Oral gavage; 574 mg/kg	6 weeks	[66]
Female growing rats	FLL ethanol extract	0.40, 0.65 and 0.90% FLL extract in AIN-93G diet	16 weeks	[67]
Female aged rats	FLL ethanol extract	Oral gavage; 700 mg/kg/d	12 weeks	[68]

## Abbreviations

1,25-(OH)_2_D_3_	1,25-Dihydroxyvitamin D3;
1-OHase	1α-Hydroxylase;
ALP	Alkaline phosphatase;
AUC_0-t_	Area under the plasma concentration-time curve from zero (0) h to time (t);
BMC	Bone mineral content;
BMD	Bone mineral density;
Ca	Calcium;
CaBP	Calcium binding protein;
CaSR	Calcium-sensing receptor;
C_max_	Maximum plasma concentration;
CO_2_	Carbon dioxide;
CT	Calcitonin;
DPD	Deoxypyridinoline;
EAF	Ethyl acetate-soluble fraction of EE;
ER	Estrogen receptor;
ERE	Estrogen receptor α/β-mediated estrogen response element;
FGF-23	Fibroblast growth factor-23;
FLL	Fructus Ligustri Lucidi;
FLL-EAF	Ethyl acetate-soluble fraction of EE;
FLL-EE	FLL ethanol extract;
FLL-WF	Water-soluble fraction of EE;
K	Drug clearance rate constant from the central chamber to the outside in the one-compartment model.
MRT	The average time of drug molecules retained in vivo after rapid intravenous infusion;
Nox4	NADPH oxidase 4;
NFATc1	Nuclear factor of activated T cell-c1;
NF-κB	Nuclear factor of kappa B;
OA	Oleanolic acid;
OCN	Osteocalcin;
OPG	Osteoprotegerin;
OVX	ovariectomized;
PTH	Parathyroid hormone;
RANK	Receptor activator of nuclear factor kappa B;
RANKL	Receptor activator of nuclear factor kappa B ligand;
ROS	Reactive oxygen species;
T_1/2_	The time taken a drug to clear from the highest concentration to half of this level;
TCM	Traditional Chinese Medicine;
T_max_	Time at maximum plasma concentration;
TRAP	Tartrate resistant acid phosphatase;
TRPV6	Transient receptor potential vanilloid 6;
UA	Ursolic acid;
VDR	Vitamin D receptor.
